# Spatial-temporal distribution of salinity and temperature in the Oued Loukkos estuary, Morocco: using vertical salinity gradient for estuary classification

**DOI:** 10.1186/2193-1801-3-643

**Published:** 2014-10-30

**Authors:** Mohamed Ali Geawhari, Lloyd Huff, Nadia Mhammdi, Athena Trakadas, Abdellah Ammar

**Affiliations:** Faculté des Sciences Rabat, Département Sciences de la Terre, Université Mohammed V – Agdal, Agdal, Morocco; Center for Coastal and Ocean Mapping, University of New Hampshire, Durham, New Hampshire USA; Institut Scientifique, Département Sciences de la Terre, Université Mohammed V – Agdal, Agdal, Morocco; Centre for Maritime Archaeology, University of Southampton, Southampton, UK; Maritime Archaeology Programme, University of Southern Denmark, Esbjerg, Denmark; Morocco Maritime Research Group, Copenhagen, Denmark

**Keywords:** Characteristics of salinity, Stage of tide, Estuary classification, Oued Loukkos, Morocco

## Abstract

**Background:**

Several different classifications to characterize estuarine systems have been proposed. In this present paper, one of the most important estuaries in North Africa, the Oued Loukkos (Morocco), forms a case-study for proposing a systematic classification of this particular tidal estuary according to the vertical salinity gradient.

**Findings:**

This study, conducted using a CTD, shows that the spatial-temporal distribution of salinity depends on the stage of the tide and the upstream distance from the mouth of the river. In this case, it is also evident that the morphology of the bottom was capable of impacting the distribution of salinity by locally changing the water circulation.

**Conclusions:**

Based on the vertical salinity gradient measurement, the Oued Loukkos represents an estuarine environment with one section near its mouth that can be characterized as a mixed mesotidal estuary and another section upstream which can be characterized as a stratified mesotidal estuary. Between, there is an intermediate zone with a low vertical gradient of salinity, classified as a partially mixed mesotidal estuary. When the effect of terrestrial inputs is low compared to marine inputs, the river bed topography plays a role in the stratification of salinity by either disrupting the vertical stratification of the water or by changing the lateral distribution of salinity. The proposed classification deepens our hydrological knowledge and provides descriptive labels to the Oued Loukkos estuary. It provides a valid starting point for predicting the environmental impact of future recreational, agricultural and commercial activities on the estuary.

## Introduction

As defined by Pritchard (1967) and Perillo (1995), estuaries in general are river systems formed inland, having one or more free connections with the sea, such that marine salts enter freely and are significantly diluted with river water. Estuaries exhibit a variety of distinguishing characteristics which have been used by various authors to describe specific estuaries and to define groups of estuaries that are similar in one, or more, important characteristic/s. Estuaries have been grouped according to: 1) topography (Pritchard 1952), 2) salt structure (Simmons 1955; Cameron and Pritchard 1963; Pritchard 1967; Dyer 1997), 3) tidal range (Davies 1964; Hayes 1975; Allen 1993), 4) propagation of the tide (Larras 1965), and 5) dominate flow regime (Dyer 1986; Dalrymple et al. 1992).

Since 1980, the estuary of Oued Loukkos, in northwest Morocco, which according to the topographic classification of Pritchard (1952) is a coastal plain estuary, has been the subject of many research programs (Snoussi 1980; Snoussi 1984; Bazairi et al. 2005; Cheggour et al. 2005; Fekhaoui 2005; Yahyaoui and Azeroual 2005; Aloussi 2008; El Morhit et al. 2008; Palma et al. 2012) that focused on defining its hydrological, sedimentological and biological organization. However, none of the previous research conducted has provided a systematic classification of the Oued Loukkos estuary, which is essential for a general understanding of the environment and of the possible impacts that future recreational, agricultural and commercial activities may have on the estuary.

As a case-study, a new hydrographic survey of the Oued Loukkos estuary was undertaken 14–24 October, 2010, and hydrological parameters were measured to assess the characteristics of the water masses throughout the estuary. Profiles of salinity and temperature were collected at different points with a CTD (conductivity, temperature, and pressure [depth] sensor). As a result, this present paper provides a systematic classification according to the salinity gradient and at the same time deepens our hydrological knowledge of the Oued Loukkos estuary, one of the most important estuaries in North Africa.

### Location of the study area

**Figure 1:**
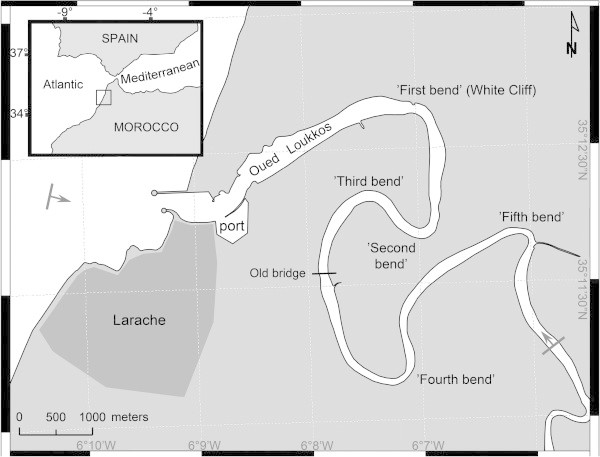
**The Oued Loukkos, Larache, Morocco.** Gray bars with arrows indicate the boundaries of the hydrographic survey.

The study area is located between 35.215 and 35.177 degrees North and between −6.17 and −6.1 degrees West (Figure 1). The Oued Loukkos, with a total length of 180 km, originates in the central Rif Mountains of Morocco and reaches the Atlantic coast at the city of Larache. The lower river, where the data for this study were obtained, is subject to semi-diurnal tides with a tidal range between 2.5 and 3 m, which is mesotidal according to the classification by Davies (1964), Hayes (1975) and Allen (1993) and is a tide-dominated system, according to the classification of Dalrymple et al. (1992).

The river and its tributaries, Oued Ouarour and Oued El Makhazine, drain a catchment of 3,750 km^2^ with an average altitude of 300 m. Since 1979, the Loukkos basin has had two large dams ensuring the development of water resources in the region of Larache: 1) the great dam of the Oued El Makhazine, whose purpose is to ensure a water supply for drinking and industrial use in urban centers, and to protect against floods, etc., and 2) the Garde dam which has a dual mission to protect the lower valley against the rise of saline water from the sea and enhance/increase the level of water in the river to facilitate pumping for irrigation (Fox et al. 1997). It should be noted that the major tributaries of the Oued Loukkos join the river above the Garde dam (located ca. 21 km upstream from the river mouth). Because the Garde dam impedes the free movement of salt further upstream, the upstream extent of the Oued Loukkos estuary ends at this dam, even though the river extends many kilometers inland beyond that point.

The climatic regime of the Loukkos basin is Mediterranean with oceanic influences. The average annual rainfall varies with altitude: 680 mm in Larache and over 1,300 mm in the mountains of the upper Loukkos valley. Typically the summer time period of low rainfall ends in late September or early October. There was no rainfall during the survey period; however, two significant rainfalls were recorded in Larache during the week preceding the present survey: 23 mm and 18 mm on 9 and 12 October, 2010, respectively^a^.

Sediment yield in the Oued Loukkos is considerable due to significant erosion in the Rif Mountains. However the sediment is generally intercepted by the great dam of the Oued El Makhazine, with the majority occurring in the rainy-season between November and March. During the month of October (this study’s measurement time), sediment intake below the Garde dam is almost nonexistent (Fox et al. 1997).

### Materials and methods

The hydrological data analyzed here were acquired during the period 14–24 October, 2010, using a SBE (Sea-Bird Electronics) CTD model 37 SMP^b^. The CTD was deployed in the Oued Loukkos estuary using a shallow-draft, 6.8 meter-long, outboard-motor powered fishing boat, *Zouhair 3*, based in the port of Larache. The boat was outfitted with a temporary enclosure to house the survey and navigational equipment. The speed of the boat and the large distances covered by the survey precluded obtaining a truly synoptic measurement of the temperature and salinity. A round-trip in *Zouhair 3*, from the mouth of the river to the most upstream reach of the survey, 15 km, and return, required a significant portion of one cycle of the semi-diurnal tide in the Oued Loukkos. The set of temperature and salinity measurements acquired between the mouth of the river and the most upstream reach of the survey consists of 103 discrete CTD casts and four continuous near-surface (0.2-0.5 m) tows of the CTD along the main axis of the estuary. Fifty-seven of the CTD casts were co-located with sites throughout the estuary where a uniform distribution of sediment samples was collected from the river bed. Taken as a whole, the CTD data adequately provide evidence to support classification of the Oued Loukkos estuary.

After acquisition, the raw numerical data from the CTD were converted into physical units for analysis and visualization. Processing was performed using Excel worksheets and oceanography “Ocean-Data-View” Software.

The continuous stages of the tide presented in this paper are nominal. They were developed using a half-cycle sinusoidal expansion technique between successive highs and lows predicted for the port of Larache. The predicted heights at high tide varied between 1.3 m and 2.2 m. The predicted heights at low tide varied between 0.0 m and 0.9 m. Neap tide was predicted to occur on 15 October. The expanded tide predictions were determined to be suitable by comparison with 28 hours of recorded CTD data in the port at 10-second intervals Geawhari et al. (2012) and a set of direct surface contact measurements at 15-minute intervals during daylight hours at the old bridge on highway N1 (Tangier-Larache Road), which is approximately 8 km upstream from the port of Larache (see Figure 1). At the old bridge, the observed range of tide was 95% of that predicted for the port. The times of high and low tide at the old bridge were delayed by 10 and 30 minutes, respectively, relative to their predicted times at the port of Larache. On 15 October, when working at the far upstream reach of the survey and near the expected time of low tide of 0.6 m, it was noted that the vertical expanse of mud exposed between the water surface and the high-tide vegetation was approximately 1.3 m. While this observation strongly implies that the tidal range at the 15 km upstream limit of the survey must be at least 85% of the predicted tidal range for the port, nothing can be implied concerning the times of high and low tides at that point, relative to the their predictions for the port, or about tidal ranges farther upstream to the Garde dam (functionally, the extreme inland reach of the estuary).

## Results

### Temperature (spatial-temporal variation)

The near-surface temperature measurements in the Oued Loukkos estuary in October 2010 varied between 18.7° and 22.5°C. Small variations in the measured near-surface temperatures, on the order of 0.1°C, were due to speed dependent variations in depth of the near-surface towed CTD measurements. Larger variations in the measured near-surface temperatures were due to changes of insolation throughout the day and changes in the stage of the tide when/where particular near-surface measurements were made.

The highest values of near-surface temperature were typically observed during the period between 11:00 and 16:00, local time. A similar observation was made by El Morhit (2009) in the same study area, and by El Kaim (1972) and El Herradi (1989) on the Oued Sebou and Oued Bouregreg, on the Atlantic coast of Morocco 115 km and 147 km south, respectively, of where the Oued Loukkos connects with the Atlantic Ocean at Larache.

**Figure 2:**
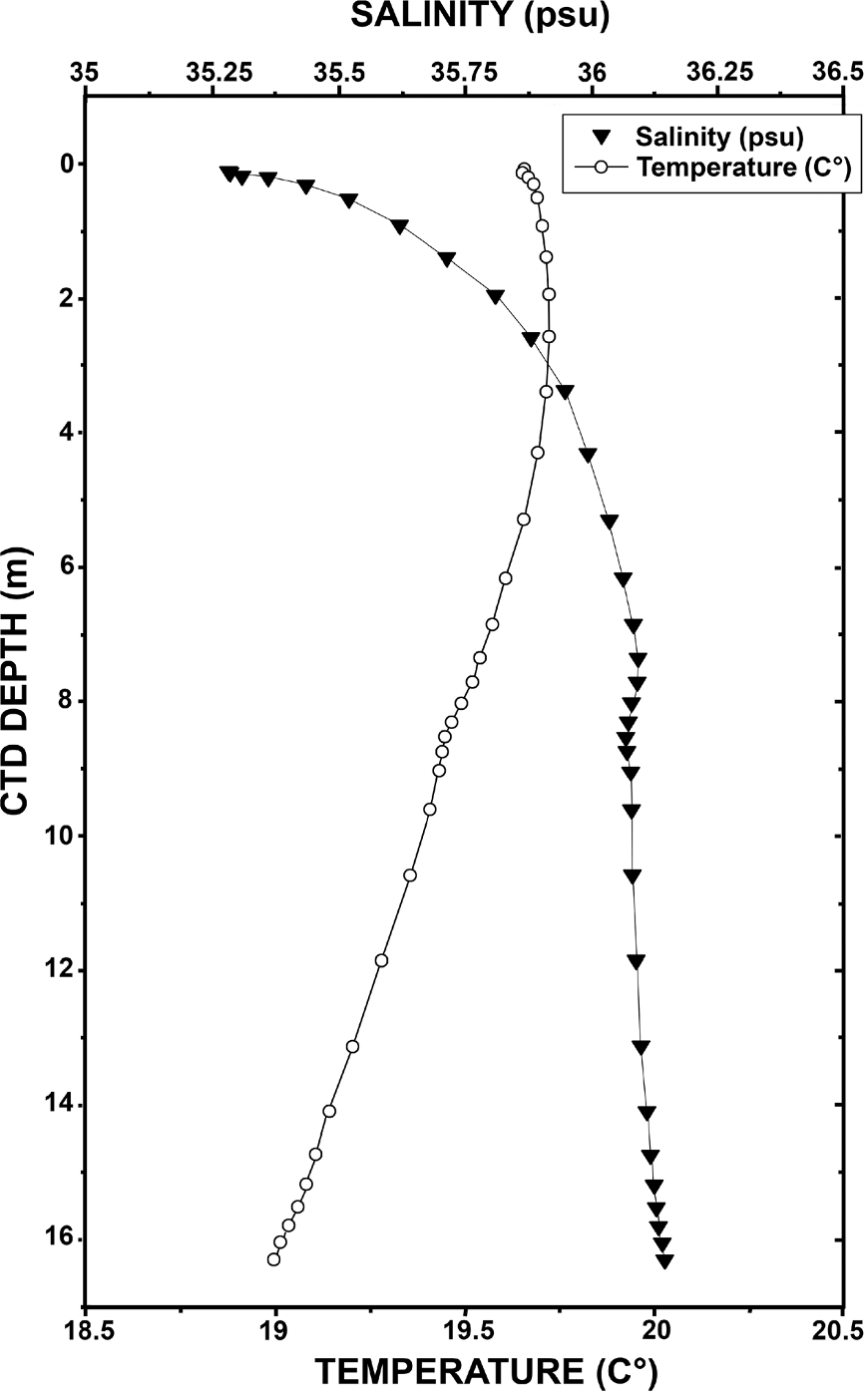
**Vertical profiles of temperature and salinity recorded on 19 October during a CTD cast taken near the predicted time of a mid-flood tide at the mouth of the Oued Loukkos.**

The temperature of the water in the estuary decreases with depth, however, only marginally. At both low and high stages of the tide, the difference in water temperature between the near-surface and near-bottom rarely exceeded 1°C. Figure 2 presents temperature and salinity profiles measured near mid-flood tide on the morning of 19 October at the mouth of the river. The character of the temperature and salinity profiles in the upper 6 m of the water column is consistent with their having recently been a surface influx of fresh water into the system which mixed with the more saline underlying water. The lowest salinity in the figure was measured at 0.27 m depth and it is possible that in the upper 2 decimeters of the water column, there may be waters whose salinities are slightly lower than any of the measured values presented in the figure.

### Salinity (spatial-temporal variation)

The salinities that were observed over all times and locations in the estuary ranged between 0.7 psu and 36.2 psu. The salinity varied between those two limits, depending on the stage of the tide and the location within the estuary. Lower values of salinity occurred in the upstream reaches of the estuary, 15 km from the mouth. Higher values of salinity occurred near the mouth of the river.

**Figure 3:**
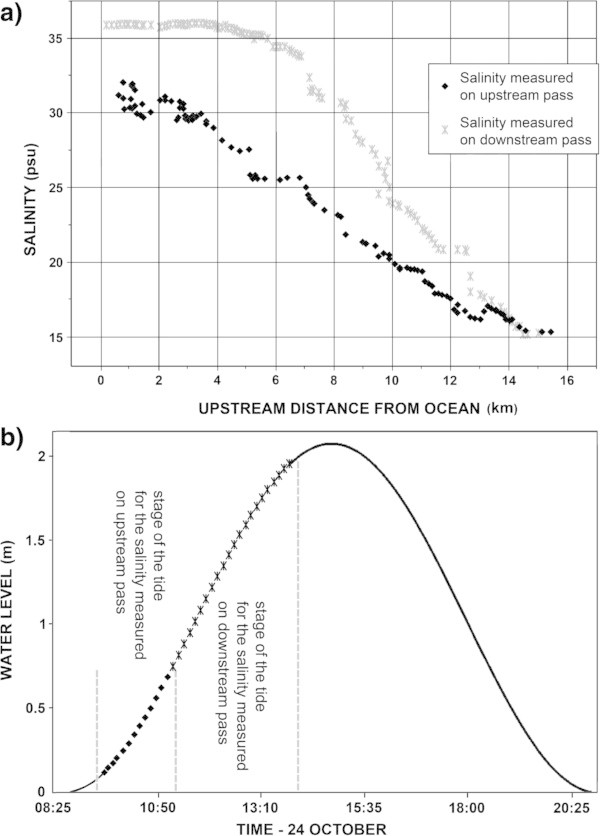
**Surface salinity variation in the Oued Loukkos. a)** Surface salinity variation as a function of distance from the ocean; **b)** Surface salinity variation as a function of stage of tide.

The near-surface salinity throughout the survey area exhibited a general horizontal gradient, with salinity decreasing upstream from the mouth of the river. The high near-surface salinity at the mouth of the river appeared to be modulated by the stage of the tide. Figure 3 shows approximately 32 psu shortly after a low tide of 0.0 m when the 24 October round-trip transect started at the mouth of the river and later shows approximately 36 psu shortly before a high tide of 2.1 m as the round-trip transect ended near the mouth of the river. On the return leg of the round-trip transect, when *Zouhair 3* was still 4 km upstream from the mouth, the near-surface salinity approached a high plateau and remained there until reaching the mouth of the river, at the end of the transect. This observation indicates that on a flood tide, near-surface water from the ocean is capable of advancing virtually unchanged for a considerable distance up into the estuary.

The low near-surface salinity at the far upstream reach of the survey also appeared to be influenced by the stage of the tide. There the variation with stage of the tide is inferred to range from 0.7 psu at low tide to something greater than 16 psu at high tide, because 0.7 psu was observed there near low tide on 15 October, 7 psu was observed there near a mid-flood tide on 17 October, and 16 psu was observed there near a mid-flood tide on 24 October.

The difference between the time required for the upstream and downstream transects (Figure 3) was due to the boat’s advance over the ground being aided by the flood tide on the upstream pass and being retarded by the flood tide on the downstream pass. When towing a CTD at very shallow depths, speed relative to the water governs the towing response of the CTD and determines whether or not air bubbles are apt to pass through the conductivity sensor and cause erroneously low salinity values. This was an important operational consideration during the downstream pass of the round-trip transect, because, although the flood tide was slowing the boat’s speed over ground, it was increasing the tow speed of the CTD relative to the water.

**Figure 4:**
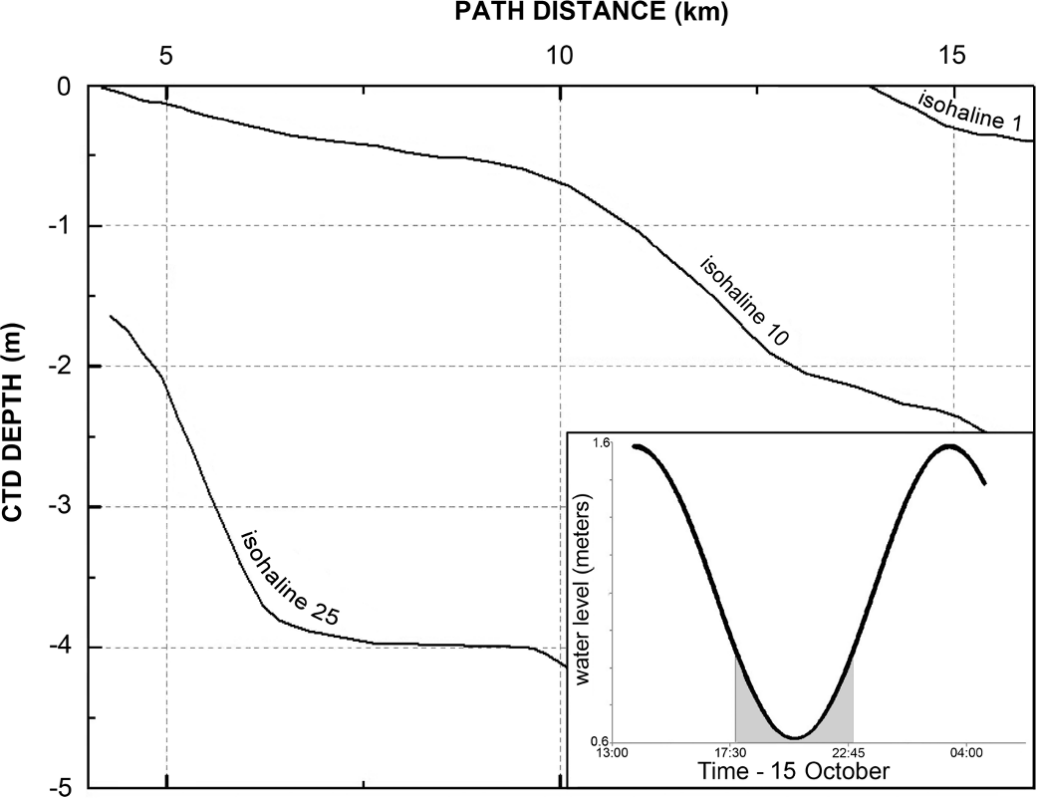
**Salinity stratification recorded during a low stage of tide in the far inland reach of the survey.**

In the vertical plane, a gradient of salinity (increasing from lower near-surface values to higher near-bottom values) was generally observed along the study area of the Oued Loukkos. The strength of the gradient varied with the stage of the tide and the distance upstream from the mouth of the river. For example, on 15 October, in the far inland reach of the survey and during an ebbing tide, fresher water was observed overlying more saline water. In this instance, the salinity ranged from 0.7 psu in the near-surface water to greater than 25 psu at a depth of 4 m, with a temperature close to 21°C throughout the entire water column (Figure 4).

CTD data acquired on a down-cast during the waning flow, approaching high tide in the vicinity of the so-called “White Cliff” (at the first bend of the river, see Figure 1) on 19 October, show that at a depth of 4.9 m, the salinity drops from 35.3 psu to 31.7 psu and then reverts to its initial near-surface value of 35.3 psu at 7 m depth. It was noted that the water surface upstream from White Cliff was pock-marked. There were large scale swirls and boiling of the upstream waters, whereas the water surface downstream was not similarly pock-marked. CTD data, acquired on the up-cast associated with the afore-mentioned down-cast, did not show a similar salinity anomaly to that observed on the down-cast. The notably pock-marked surface led to an interpretation that the cited salinity anomaly was due to the difficulty of adequately sampling the temporal/spatial variability of a turbulent flow, rather than being due to temporary malfunction of the CTD’s conductivity sensor. CTD data acquired during slack water at high tide on 20 October, in the vicinity of White Cliff, show salinities that were greater than 35.5 psu over the entire water column and neither the upstream nor downstream water surfaces were pock-marked.

## Discussion

### The influence of bottom topography on the distribution of salinity

According to previous studies (Snoussi 1980) and our field observations, starting shortly upstream of the entrance to the port of Larache, the channel of the Oued Loukkos is characterized by depths that generally vary between 2 and 4 m below chart datum, which is approximately Lowest Astronomical Tide (LAT). However, ca. 3.5 km upstream from the mouth of the river, at the White Cliff bend, there is a significant change in the depth of the river, to 18 m; a short distance after that, the depth quickly reverts to varying between 2 and 4 m. This “pit” at the White Cliff is a unique and remarkable feature in the river bed morphology of the Oued Loukkos.

**Figure 5:**
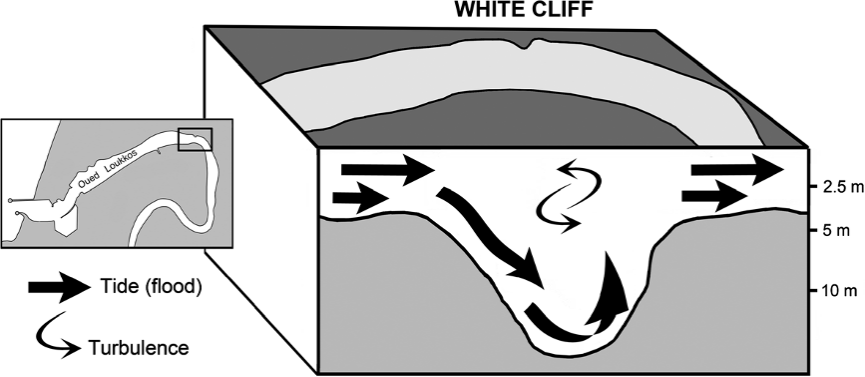
**Conceptual influence of the pit at White Cliff on the Oued Loukkos’ hydrodynamic flow during a flood tide.**

The White Cliff pit in the river bed apparently causes changes in the local water circulation. In this area of the river, it is possible that the vertical structure of the salinity field is impacted by strong dynamic effects that can override the weaker static effects of the vertical density structure. Figure 5 illustrates a conceptual view of vertical mixing during a flood tide whereby convection cells at the White Cliff maintain the pit by flushing out any fine sediment that may settle there during slack water and support the temporary/dynamic inclusion of water at a given depth with a density (salinity) that would not have occurred under static conditions. If the conceptual view were to be of vertical mixing during an ebb tide, the figure would be the same, except the direction of the flow vectors would be reversed.

The White Cliff pit probably influences the salinities in the estuary at considerable distances, both upstream and downstream, from the pit. The transects of near-surface salinity (see Figure 3) exhibit a change in their character in the vicinity of the White Cliff pit, when approaching it from both the downstream and upstream directions. The pit possibly exerts an influence on salinity gradients due to partial conversion of the energy of the tide into turbulence. The interaction of the tidal flow with this morphological peculiarity of the river bed may explain the upstream presence of a significant salt gradient.

### Classification of the estuary

Salinity and temperature determine the density of water and variations in density may lead to vertical movement of water masses. Vertical variations in temperature and/or salinity are expected to lead to upward movements for water that is less dense (fresher and/or warmer), than the overlaying water and downward movements for water that is more dense (colder and/or more saline) than the underlying water. In our study area, the vertical temperature gradients were low; therefore temperature most likely plays a minimal role in the density stratification. Differences between the salinity of surface and bottom waters can be large, therefore the vertical density structure in the Oued Loukkos estuary is most likely determined by salinity.

As previously referenced, there are several classification schemes that may be applied to the characterization of an estuary, including ones based on the tidal range, the relationship between the effects of river flow and the tide, and stratification of saline waters.

**Figure 6:**
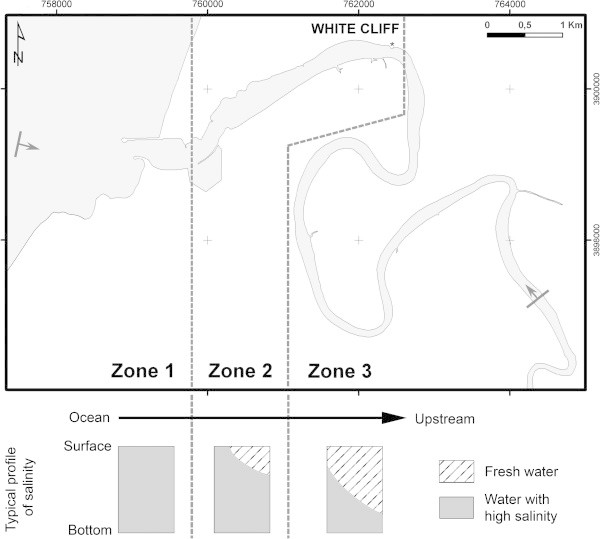
**Subdivisions of the Oued Loukkos based on the vertical gradient of salinity, with the typical profiles of salinity for each zone.** Gray bars with arrows indicate the boundaries of the hydrographic survey.

We propose subdividing that portion of the Oued Loukkos estuary, which lies between the mouth of the river and 15 km upstream from the mouth, into three zones that are based on characteristics of the vertical salinity gradient (Figure 6). (Additional surveying would be required before any informed comment could be offered concerning the ca. 6 km upstream portion of the Oued Loukkos estuary that lies above 15 km and extends to the Garde dam.)

Zone 1: Vertical salinity gradients are very low and the zone is also characterized by a high salinity. In this area, between the mouth of the river and the entrance to the port of Larache (ca. 1 km), the tidal oscillation of water masses, incoming ocean swells (El Morhit 2009), and possibly the effect of a seiche phenomenon that can be present in the port of Larache (Geawhari et al. 2012), promote mixing within the water column.

Zone 2: Vertical salinity gradients are low and the zone is also characterized by high mean salinity and moderate tidal variability of salinity. This zone has a length of 3.5 km and is the part of the river between the port of Larache and the first significant bend of the river, at White Cliff. The tidal cycle, acting in conjunction with the unique bathymetric feature at White Cliff, causes vertical exchange between fresh water and salt water.

Zone 3: Vertical salinity gradients are large and the zone (from ca. 4.5 km upstream) is also characterized by fresh surface waters being enriched with salt toward the downstream direction, while bottom waters tend to freshen heading in the upstream direction. The existence of a large vertical gradient of salinity in this area may lead to trapping and concentration of suspended sediment to create a turbidity maximum.

Because of gaps in time and space in the 103 CTD casts and four continual transects, comprehensive representation of the spatial-temporal evolution salinity and temperature fields in the Oued Loukkos estuary has been difficult. However, it is clear that the characteristics of salinity in the river vary considerably between good homogeneity of salt near the mouth of the river and a significant stratification in the far inland reaches of the river. Near the mouth of the river there is no distinct halocline, or thermocline, while upstream, and especially after the third bend in the river, the differences between surface and bottom salinity can be very high, as evidenced by the presence of a distinct halocline.

By combining the subdivision that we propose with different classifications from the literature, one can make a further deduction about each of the three zones.

Zone 1 shows the characteristics of a mixed mesotidal estuary with practically low vertical salinity stratification.

Zone 2 shows the characteristics of a partially mixed mesotidal estuary. The complex topography of the river bed in this area promotes turbulence and impacts the vertical water stratification.

Zone 3 shows strong vertical stratification. This type of stratification is observed mainly in microtidal estuaries (tide range <1 m) (Davies 1964; Hayes 1975; Allen 1993), although in this part of the estuary the Oued Loukkos retains its mesotidal property, which qualifies this area of Oued Loukkos as a well stratified mesotidal estuary. This implies that when the flow in the river is low, as it was during this survey, the effect of the bed topography lengthens the residence time of fresh water in the river, and thereby plays a role in freshening the water in this upstream reach of the estuary.

## Conclusions

This review of the factors that influence the waters between the mouth of the Oued Loukkos and 15 km upstream has highlighted salinity-related marine influences in the estuary, and it is proposed that classification of the estuary should focus on its salinity gradient.

The important characteristics of the salinity distribution in the Oued Loukkos are: 1) variations along the main axis of the estuary exhibit an along stream gradient with salinities decreasing as distance increases upstream from the mouth, and 2) vertical variations which exhibit a greater degree of homogeneity near the mouth of the river, compared to that upstream.

Thus, according to the degree of homogeneity along the Oued Loukkos, we conclude that the reach of the estuary near the mouth of the river has the characteristics of a mixed estuary and after passing through an intermediate zone with the characteristics of a partially mixed estuary, the upstream (inland) reaches have the characteristics of a stratified estuary. The tidal characteristic throughout the entire Oued Loukkos estuary is mesotidal. At those times when the effect of terrestrial inputs is low compared to marine inputs, the river bed topography probably plays a role in the stratification of salinity by either disrupting the vertical stratification of the water or by changing the lateral distribution of salinity.

In the opinion of the authors, the importance of these conclusions go far beyond that of having applied appropriate descriptive labels to the Oued Loukkos estuary, because it provides a valid starting point for predicting the environmental impact of future sustainable undertakings in the Oued Loukkos estuary, including recreational, agricultural and commercial activities.

## Endnotes

^a^For rainfall data see, http://www.infoclimat.fr/climatologie/globale/12-octobre/larache/60105.html; accessed 9/2014.

^b^Acquired conductivity, temperature, and pressure (depth) data were stored on an internal 64 MB flash card and later transmitted in ASCII to an external computer for processing and analyses. The instrument accuracies are 0.002°C for temperature, 0.0003 S/m for conductivity and 0.1% of full scale range for depth, with resolutions that are one-percent of the accuracies (http://www.seabird.com/products/spec_sheets/37smpdata.htm; accessed 5/2014).
